# Antimicrobial and Cytotoxic Effect of Positively Charged Nanosilver-Coated Silk Sutures

**DOI:** 10.1021/acsomega.4c01257

**Published:** 2024-04-08

**Authors:** Diego Antonio Monroy Caltzonci, Aruna-Devi Rasu Chettiar, Verónica Campos Ibarra, Latha Marasamy, Marcos Loredo-Tovías, Laura Susana Acosta-Torres, Ravichandran Manisekaran

**Affiliations:** † Interdisciplinary Research Laboratory (LII), Nanostructures and Biomaterials Area, Escuela Nacional de Estudios Superiores Unidad León, Universidad Nacional Autónoma de México, Predio el Saucillo y el Potrero, Comunidad de los Tepetates, 37689 León, Mexico; ‡ Facultad de Química, Materiales-Energía, Universidad Autónoma de Querétaro, 76010 Querétaro, Mexico; § Área de Ciencias de la Tierra, Facultad de Ingeniería,UASLP, Av. Manuel Nava no.8, Zona Universitaria, 78290 San Luis Potosí, Mexico

## Abstract

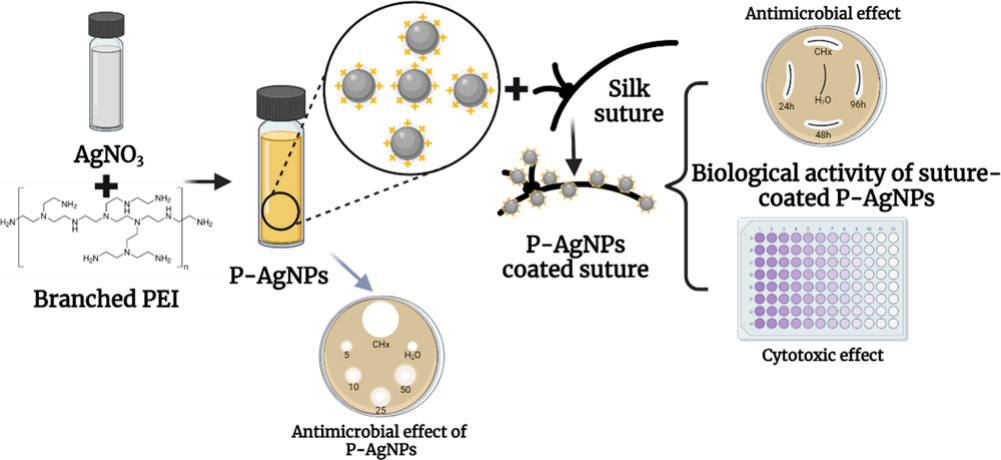

Sutures are a crucial component of surgical procedures, serving to close and stabilize wound margins to promote healing. However, microbial contamination of sutures can increase the risk of surgical site infections (SSI) due to colonization by pathogens. This study aimed to tackle SSI by synthesizing positively charged silver nanoparticles (P-AgNPs) and using them to produce antimicrobial sutures. The P-AgNPs were reduced and stabilized using polyethylenimine (PEI), a cationic branched polymer. The physiochemical characteristics of P-AgNPs were confirmed from the surface plasmon resonance (SPR) peak at 419 nm, spherical morphology with a particle size range of 8–10 nm, PEI functional groups on NPs, a hydrodynamic diameter of 12.3 ± 2.4 nm, and a zeta potential of 31.3 ± 6 mV. Subsequently, the surfaces of silk sutures were impregnated with P-AgNPs at different time intervals (24, 48, and 96 h) using an ex situ method. Scanning electron microscopy (SEM) and tensile strength studies were conducted to determine the coating and durability of the NP-coated sutures. The NPs were quantified on sutures using inductively coupled plasma optical emission spectrophotometry (ICP-OES), which was in the range of 1–5 μg. Primarily, antimicrobial activity was studied using three microorganisms (*Candida albicans*, *Streptococcus mutans*, and *Staphylococcus aureus*) for both P-AgNPs and suture-coated P-AgNPs using the agar diffusion method. The results showed that only the NPs and NP-coated sutures exhibited enhanced antimicrobial effects against bacteria and fungi. Finally, the cytotoxicity of the sutures was investigated using stem cells from the apical papilla (SCAPs) for 24 h, which exhibited more than 75% cell viability. Overall, the results indicate that NP-coated sutures can potentially be used as antimicrobial sutures to diminish or inhibit SSI in postoperative or general surgery patients.

## Introduction

The term “suture” has its roots in Latin and signifies that the use of suture materials in surgical operations has a long history. In the past, research has revealed that various materials have been used for sutures and ligatures, such as plant fibers, leather strips, bovine intestine, animal tendons, braided hair, gold wires, and other metals.^1^ Surgical suturing is an indispensable component of many surgical operations, and its primary objectives include wound closure, tissue margin reinforcement, and promotion of healing.^2^ Sutures can be categorized based on their properties as absorbable or nonabsorbable and their origin, whether natural or synthetic. Additionally, sutures can be classified as monofilament or multifilament based on the number of filaments used.^3^ The suture is usually inserted into highly vascularized tissues, and when teamed with moisture, it creates a suitable environment for the growth of infectious microorganisms.^4,5^ Therefore, the contamination of surgical materials is a significant risk factor for infection, accounting for 20% of healthcare-associated infections. Microorganism adhesion is influenced by various suture characteristics.^6^

Surgical silk- and polymeric-based sutures are widely used in a variety of surgical procedures. Silk is a biological material commonly composed of intertwined or braided fibers. Modern silk is known for its softness and ease of handling, which makes it the preferred choice for suturing. It has been widely recognized for its utility in a range of medical procedures including dental, ocular, neural, and cardiovascular surgery. This is because of its ease of handling, which makes it unsuitable for other materials. Despite its longer duration of use, the degree of bacterial infection associated with silk sutures is still lower than those associated with other materials. However, its tensile strength is lower than that of other materials and decreases over time, with complete absorption occurring within two years.^7^

As the field of nanotechnology progresses rapidly, various nanomaterials often display unique and considerably modified physical, chemical, and biological properties compared with their macroscale counterparts. This concept has attracted the interest of scientists owing to its exploitation in diverse applications.^8^ Among many other metals, noble metal silver (Ag) is commonly employed and studied owing to its exceptional properties, including high conductivity, chemical stability, and catalytic and antimicrobial capabilities. This makes AgNPs effectively incorporated into various biomedical applications, such as antimicrobial, biosensing, drug delivery, tissue regeneration, implants, dressings, cosmetics, and coatings.^9^ AgNPs are known for their remarkable inhibitory and microbial effects on bacteria, fungi, parasites, and viruses.^10^ The broad spectrum of action or mechanism depends on the release of silver ions that produce ROS and toxic agents that can bind to membrane proteins and nucleic acids, causing a cascade of actions, including structural changes, denaturation, and inhibition of replication, leading to cell death.^11^

Many studies have been carried out using AgNPs against microbes, including COVID-19.^12^ In recent years, efforts have been made to reduce postsurgical infections using various alternatives, including the use of AgNP-coated medical devices. In this context, some studies have investigated AgNP-based antimicrobial sutures against several pathogenic microorganisms. Dubas et al. evaluated the antimicrobial activity of sodium alginate-based AgNPs in sutures and demonstrated an enhanced antimicrobial effect.^13^

Zhang et al. coated AgNPs on the surface of absorbable sutures to explore their anti-inflammatory efficacy and potential clinical applications in an intestinal anastomosis model.^14^ Baygar et al. designed a study to coat silk sutures with AgNPs using green synthesis and evaluated their compatibility, cytotoxicity, and antimicrobial effects.^15^ Syukri et al. biosynthesized AgNPs using *Eucalyptus camaldulensis* and coated sutures, thereby elucidating a strong bacteriostatic effect on *Staphylococcus aureus* and other Gram-negative bacteria’s with 99% reduction for up to 12 weeks with 80% cell viability.^16^ In 2021, the same group covered nylon sutures with AgNPs and demonstrated bactericidal effects on Gram-positive and Gram-negative wound pathogens, with a reduction of >99.9%.^17^

In recent studies published by Selvaraju et al., 20 nm AgNPs reduced by propolis have been used to coat catgut sutures and the antimicrobial effect of clinical pathogens. The results showed that NPs exhibited enhanced antimicrobial effects, thereby preventing SSI-based pathogens.^18^ Based on our search, there are very few investigations in this area; therefore, in this work, we developed highly cationic P-AgNPs using polyethylenimine (PEI) as both a reducing and stabilizing agent without using any extra reagents. Our previous study on various *Candida* species revealed that NPs exhibited exceptional antifungal activity.^19^

Thus, NPs were used to coat silk sutures with NPs using an ex situ method. Additionally, the physicochemical properties of the suture-coated NPs were investigated. To determine the potential synergistic effect of antimicrobial sutures, biological effects, such as antimicrobial activity, were studied against bacterial and fungal microbes. Finally, the cytotoxic behavior of the suture was evaluated to understand the cellular interactions.

## Materials and Methods

All chemicals and solvents used in this study were procured from Sigma-Aldrich (Mexico), unless otherwise mentioned.

### P-AgNPs Synthesis

P-AgNPs were synthesized by using a chemical reduction method. The protocol was similar to that of a previously published methodology with minor modifications.^19^ For the synthesis, 10 mM silver nitrate (AgNO_3_, ≥99%) was boiled under constant stirring. At this point, 10 mM branched polyethylenimine (40 μL, PEI-H(NHCH_2_CH_2_)_*n*_NH_2_, *M*_w_ ∼ 25,000) was added to the silver precursor and allowed to change color (colorless yellow) for 20 min. The final colloidal solution was precipitated using acetone to obtain a pellet, which was redispersed in water and refrigerated.

### Preparation of Sutures

For this study, we used silk sutures purchased from a dental store in Leon, Mexico. Specifically, silk suture of 3–0 black braided nonabsorbable (75 cm, Matsed, Matcur, Mexico) was used. Initially, 2.5 cm (∼3 mg) and 1.5 cm (∼1.6 mg) fragments were obtained, cleaned in ethanol (≥99.5%) suspension, and placed in a shaker (Digital Orbital Shaker, Heathrow Scientific) for 24 h.^20^ Subsequently, excess alcohol was removed, and the sample was placed in a heated oven for 24 h at 25 °C. This ensured that the sutures did not contain any organic content or coating during the fragmentation or handling process.

### Coating of Sutures with P-AgNPs

The prepared suture fragments were immersed in the as-synthesized colloidal P-AgNP suspension, and the tubes were maintained under shaking conditions and left to soak for 24, 48, and 96 h to ensure impregnation of the suture surface. Following each interval, the excess suspension was removed from the tubes, and the fragments were rinsed with distilled water to remove loosely bound NPs, which were then placed in an oven for 24 h at 35 °C to completely dry.

### Characterization Techniques

#### P-AgNPs

The optical properties were studied by UV–visible spectroscopy (Multiskan GO, Thermo Scientific). The morphology was determined by transmission electron microscopy (TEM; JEM 1010, JEOL). The functional groups were observed by using Fourier-transform infrared spectroscopy (FTIR, PerkinElmer Frontier). The average particle size and zeta potential were analyzed by using a Zetasizer Nano ZS90 (Malvern Panalytical, Malvern, UK).

#### NPs-Coated Sutures

The surfaces of the sutures coated with P-AgNPs were analyzed using scanning electron microscopy equipped with energy-dispersive X-ray spectroscopy (SEM-EDS, HITACHI TM1000). The mechanical properties of the NPs coating on the sutures were evaluated by using a tensile tester with a force/torque indicator (Mecmesin). The sutures were subjected to traction until they broke, and measurements were recorded in megapascals (MPa) by dividing the maximum load (in Newtons). The NPs on the sutures were quantified by using ICP-OES (iCAP 7000 series, Thermo Scientific) via microwave-assisted acid digestion. The suture fragments were weighed in a Teflon container, and 3 mL of nitric acid (HNO_3_) was added to dissolve the samples in a microwave system (Mars 6, CEM) using the United States Environmental Protection Agency (USEPA) 3051 method. Subsequently, the samples were filtered and weighed. Quality assurance was performed using reference materials, blanks, calibration curves, and analysis.

### Antimicrobial Evaluation of P-AgNPs and Suture-Coated P-AgNPs

The antibacterial and antifungal activities of the P-AgNPs and NP-coated sutures were evaluated in *S. mutans* (clinical isolate), *S. aureus* (clinical isolate), and *C. albicans* (ATCC 90028) using the agar well diffusion method. Mueller-Hinton (MH) and Sabouraud Dextrose (SD) agars were used for bacterial and fungal cultivation. The plates were filled with 20 mL of the respective agar and allowed to solidify in a laminar flow hood. Based on overnight culture, the 0.5 McFarland scale was adjusted using a sterile saline solution in the densitometer (DEN-1B, Grant Instruments). Subsequently, 80 μL of the microbial solution was dropped onto the prepared plates using 8–10 glass beads for uniform growth. Later, using a sterile 1 mL pipet tip (8 mm), the well was filled with 100 μL of NPs of various concentrations (5, 10, 25, and 50 μg) along with standard 2% chlorhexidine (CHx-25 μg, FGM, Mexico) and distilled water as a control. The plates were incubated at 37 °C for 24 h. The zone of inhibition (ZOI) was measured using a Vernier caliper, and photographs were taken.^21−23^ In the case of sutures, the exact procedure mentioned above was carried out, but instead of a well, 5 cm sutures were placed in the agar plates using sterile tweezers and incubated for 24 h, after which the ZOI was measured.

### Cytotoxic Studies

To assess the cytotoxic effects of the suture-coated P-AgNPs, a cell viability assay was performed using SCAPs. SCAPs cells were seeded at a concentration of 4.5 × 10^5^ cells/mL in a 96-well plate (Costar) and incubated for 24 h at 37 °C with 5% CO_2_ and 95% humidity supplemented with minimum essential medium (MEM), 10% fetal bovine serum, 1% penicillin–streptomycin, and 1% glutamine. In the present study, 1.5 cm suture fragments were inoculated, as well as sutures treated with CHx. After 24 h, the medium was replaced with the MTT reagent at a concentration of 0.002 mg/mL stock dissolved in MEM, and the mixture was incubated for 7 h at 37 °C in a CO_2_ incubator (Binder, Tuttlingen). The formazan crystals were dissolved in dimethyl sulfoxide (DMSO), and the absorbance was measured in triplicate using a microplate reader at a wavelength of 570 nm. All assays were performed in triplicates, each of which was repeated three times (*n* = 9).

## Results and Discussion

The use of AgNPs in biomedical applications has become inevitable and has been extensively investigated owing to their outstanding antimicrobial, anticancer, and optical properties.^24−26^ The particles synthesized by using diverse reducing agents ultimately bear negative charges and are not colloidal in nature. As a result, various studies have employed postsynthesis external agents to modify the surface charge of NPs. In this study, we aimed to determine the optimal PEI concentration for reduction and stabilization by leveraging amine groups. Given that most microorganisms possess a negative charge, the interaction of positively charged NPs can lead to improved interactions and internalization. Therefore, surface charge can be utilized to enhance antimicrobial action against a range of pathogens with a minimal concentration of NPs, thereby minimizing cytotoxicity.^27,28^

In this study, we attempted to reduce postoperative infections by synthesizing P-AgNPs to coat silk sutures and evaluating their biological effects on microorganisms and cells. Silk sutures were used as the model suture material because they are among the most commonly used materials in oral and other surgeries. In addition, they are composed of multifilament and nonremovable surfaces, which make them more vulnerable to microbial attachment and induce biofilm growth at the infection site. Thus, the developed antimicrobials must have a prompt, robust, and broad biocidal scale with prolonged effects without compromising biocompatibility without altering the wound-healing process without systemic absorption.

The novelty of this work is the use of PEI alone for the synthesis of AgNPs, thereby coating the suture. There are very few investigations in which only PEI was used, and in most cases, ascorbic acid,^29,30^ sodium hydroxide, sodium borohydride,^31,32^ and formaldehyde were used. In other processes, after NPs synthesis, PEI is used to change the surface charge according to the application. Particle size and surface charge play a vital role in biological applications; thus, the main aim is to synthesize smaller cationic particles, which can have a significant impact on the antimicrobial field synergistically as PEI itself has antimicrobial activity, so combining both can be more efficient in reducing microbial growth.

### P-AgNPs

**Figure 1:**
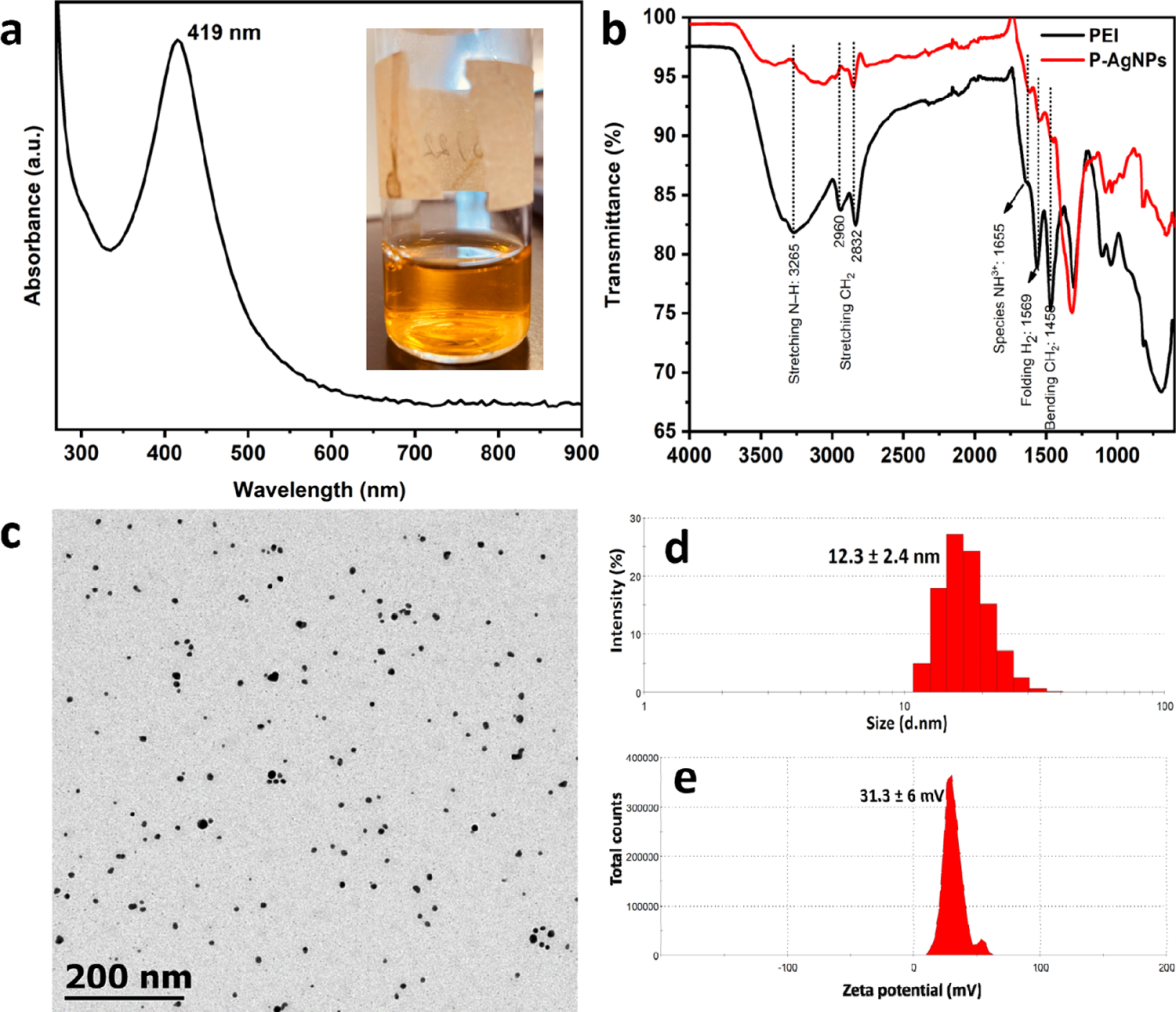
P-AgNP characteristics determined by the (a) SPR peak at 419 nm (inset: NP solution), (b) PEI and P-AgNP functional groups, (c) TEM micrograph in the range of 8–11 nm, (d) hydrodynamic size of 12.3 nm, and (e) zeta value of 31.3 mV.

The main objective of the UV–vis analysis was to determine the complete reduction of the Ag precursor and ensure the formation and stabilization of the AgNPs. During the synthesis procedure, visible observation from colorless to yellow confirmed the formation of NPs (Ag^+^ - Ag^0^). In addition, the formed solution was highly colloidal as it did not form any precipitates. The UV–vis spectrum showed a single intense absorption peak at 419 nm, which corresponds to the SPR band of the AgNPs. These data corroborate other reports that match chemical^33^ and biological Ag reduction.^34^ This result indicates that the synthesized NPs are highly spherical, as shown in Figure 1a. The mechanism behind the synthesis is the cleavage of amine groups from PEI at the boiling temperature. The liberated monomer forms Ag seeds, initiating the nucleation process and ripening, resulting in the formation of stable NPs with a positive charge.

In Figure 1b, the infrared spectroscopy analysis reveals the characteristic functional groups of the PEI polymer and P-AgNPs, including the stretching vibrations of the N–H amino group at 3265 cm^–1^, CH_2_ stretching vibrations at 2960 and 2832 cm^–1^, NH^3+^ species at 1655 cm^–1^, NH_2_ bending vibration at 1569 cm^–1^, and CH_2_ bending vibration at 1458 cm^–1^.^35,36^ The presence of these peaks was observed in both spectra; however, in the case of P-AgNPs, the intensity of PEI diminished, which corroborates that PEI was involved in the reduction and indicates successful coating on the surface of AgNPs, thereby helping in stabilization by electrostatic interactions, which prevents the aggregation of particles.^37^

The morphology and size of the NPs were observed by using TEM (Figure 1c). From the micrographs, it was proven that the particles were spherical and homogeneous, with the size distribution of the NPs ranging from 8 to 11 nm.^38,39^ The antimicrobial action was indirectly proportional to the NP size. It is well-known that the microbe size of either bacteria or fungus is in the order of microns, so when the NPs size is smaller, it enhances the internalization when coming in contact with the cellular membrane which in turn can have an increased growth inhibition

Subsequently, the size distribution was measured using dynamic light scattering (DLS) (Figure 1d), where the average particle size obtained was slightly larger (12.3 ± 2.4 nm) because the equipment measured the hydrodynamic size of the NPs, that is, when the NPs were present in the liquid medium. Subsequently, surface charge measurements were performed by using the zeta potential. The zeta value of the NPs was positive (31.3 ± 6 mV) due to the presence of significant amino groups that were exposed on the NPs surface.^32,40^ This confirmed that the PEI chains stabilized the NPs electrostatically as presented in Figure 1e.

### NPs-Coated Sutures

**Figure 2:**
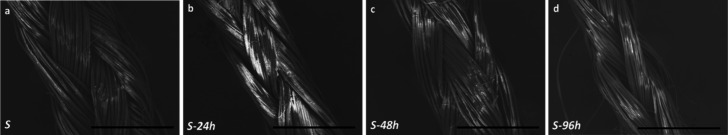
SEM analysis of the control (a) and suture coated with NPs at various time intervals (b–d) (scale bar: 300 μm).

To confirm the impregnation of P-AgNPs onto the silk suture, we first analyzed it using SEM-EDS, as shown in Figure 2a–d. The distribution of Ag particles on the surface was observed, and a typical braided structure of the suture filament was observed in the control samples without AgNPs.^41^ Under a microscope, the coated sutures showed the presence of particles. The intensity changed depending on the incubation time. Therefore, EDS-based chemical composition was studied to specifically identify the presence of Ag, which showed a heterogeneous distribution of particles on the material surface.^15,18,42^ The quantitative data are expressed in atomic % of Ag deposition as follows: compared with 48 h (24.8) and 96 h (7.3) of incubation time, the 24 h (32.2)-treated suture fragment showed more adsorption of NPs, which may be due to the supersaturation point or prolonged incubation time leading to desorption of particles under shaking conditions.

**Figure 3:**
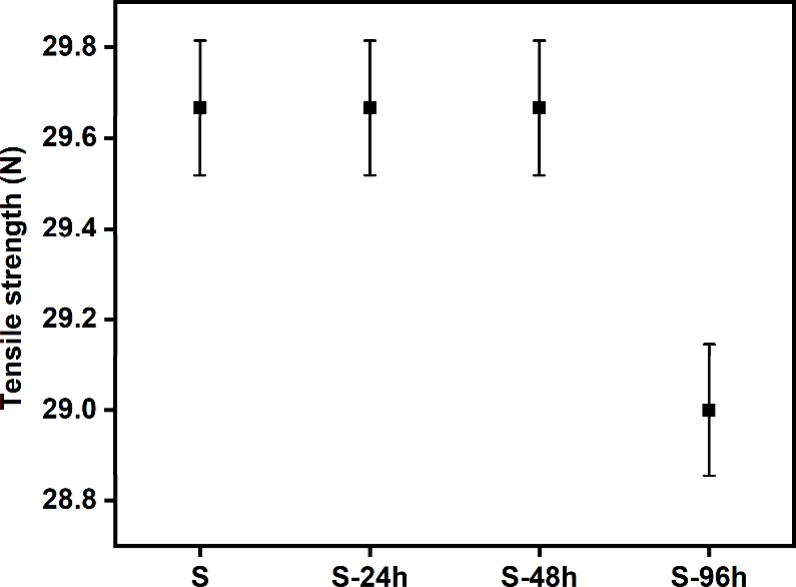
Tensile strength of the sutures and their resistance compared with the control group, which is expressed in N.

As the suture is essential for wound closure, evaluation of its resistance is very important. Therefore, the mechanical impact of particle coating on the sutures was studied using a universal tensile tester (Figure 3).^43^ Interestingly, the sutures did not exhibit any major changes in their resistance, which is similar to that reported in another study.^16^ In addition, Altuntas et al. subjected AgNP-modified absorbable sutures to modification with NPs and found that the sutures they treated had no changes in their mechanical properties^44^ at coating times of 24 and 48 h, respectively, when compared with the control sample. However, there was a minor decrease in the resistance of the sutures coated for 96 h, which was negligible, as it would not cause any severe effects.

Finally, in the present study, mass spectrometry analysis was performed using ICP to quantify the precise concentration of NPs impregnated on silk suture threads.^45^ The fragments incubated for 24 and 48 h showed 5.2 and 4.3 μg of AgNPs for fragments weighing approximately 1.5 mg, respectively. However, as seen from the above measurements, it corroborates the data where the 96 h fragment exhibited only 1.4 μg, which is 22 and 25% less than that at 24 and 48 h. This quantification directly affects the biological properties of fragments when they encounter microorganisms or cells.

### Antibacterial and Antifungal Effects of P-AgNPs

**Figure 4:**
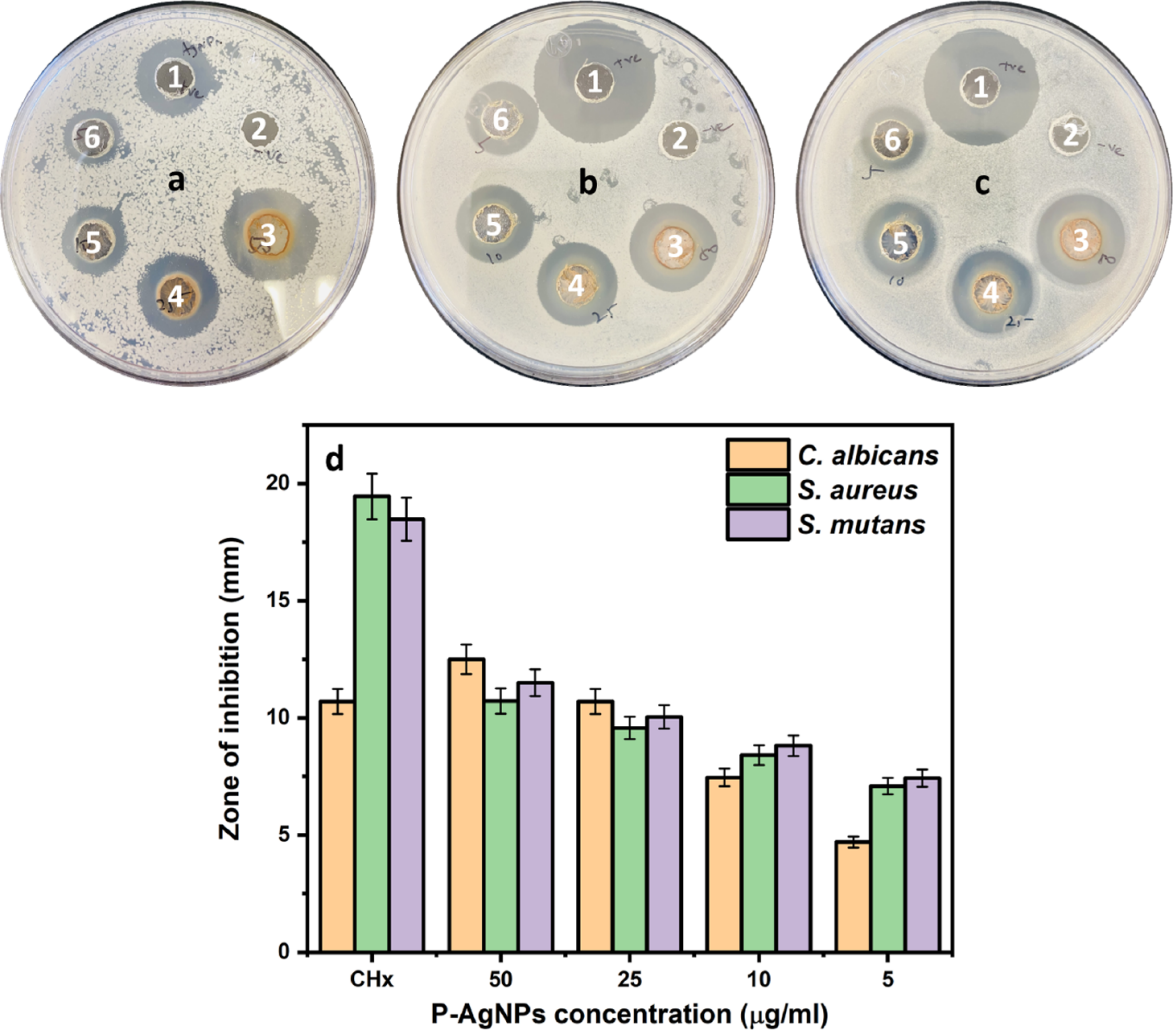
Antimicrobial effects using the agar well diffusion method. (a) *C. albicans*, (b) *S. aureus*, and (c) *S. mutans*, where 1. CHx, 2. H_2_O, 3. 50, 4. 25, 5. 10, 6. Five of the various concentrations. (d) ZOI comparison data for all microorganisms (in mm).

Before evaluating the antimicrobial effect of the suture, we studied the antibacterial and antifungal effects on Gram-positive bacteria and *Candida* species using the agar well diffusion method, as shown in Figure 4a–c. From these images, it is quite interesting that the P-AgNPs exhibited enhanced inhibition, as observed in the ZOI. Although the NPs concentration was minimal 5–50 μg, susceptibility was observed in a dose-dependent manner. CHx (25 μg) inhibited the maximum growth of bacteria, except for *Candida*, which ranged from 10.7 to 19.45 mm depending on the microbial species. An effect was observed for P-AgNPs based on the concentration. When there was a high concentration of 50 μg, *Candida* was inhibited more than bacteria with a ZOI of 10.72–12.5 mm, and as the concentration decreased from 25, 10, to 5 μg, the ZOI values of 9.57–10.7, 7.46–8.81, and 4.7–7.43 mm, respectively, were obtained (Figure 4d and Table 1). In Table 2, we have compared some of the investigations that employed positively charged Ag-based nanostructures, which have evaluated their antimicrobial effects on microbial species. Various investigations have shown that the effect against bacteria and fungi is closely similar to our results but purely based on the reducing agent, concentration, size, and type of species. This confirmed that P-AgNPs can restrict the growth of various microbial species, but they could depend on various crucial factors.

**Table 1:** Numerical Values of ZOI (in mm)

**samples (in μg)**	**ZOI (in mm)**		
	***C. albicans***	***S. aureus***	***S. mutans***
CHx-25	10.7 ± 0.535	19.45 ± 0.9725	18.48 ± 0.924
50	12.5 ± 0.625	10.72 ± 0.536	11.5 ± 0.575
25	10.7 ± 0.535	9.57 ± 0.4785	10.04 ± 0.502
10	7.46 ± 0.373	8.41 ± 0.4205	8.81 ± 0.4405
5	4.7 ± 0.235	7.09 ± 0.3545	7.43 ± 0.3715

**Table 2:** Comparison of AgNPs-Based Nanostructures and Their Antimicrobial Effect^a^

**nanostructures**	**reducing agent**	**size (nm)**	**surface** charge (+mV)	**microbial species**	**ZOI or MIC**	**references**
AgNPs	*Corallina officinalis* & NChit	64.59	34.3	*S. aureus*	19.2 ± 2.1 mm	(46)
				ATCC 25923	42.5 μg/mL	
AgNPs	*Codium capitatum* & f-Chit	79.6	21.9	*S. aureus*	23.8 ± 1.4 mm	(47)
				ATCC 43300	4 μg/mL	
AuAgNCs	BSA	8.1	37.9	*S. aureus*	5 μg/mL	(48)
AgNCs	bPEI	2	30	*S. aureus*	0.015 and 0.06 nM	(40)
				(CD489 & 1578)		
AgNPs	bPEI	7.5	42	*S. mutans*	5 μg/mL	(49)
AgNCs	Chit	2	35.9 ± 3.9	*S. aureus* ATCC 25923	10 ± 1 mm	(50)
AgNPs	bPEI	5–35	20.5–54	*C. albicans (ATCC 90028, 60804)&* Cat52 V	8.33–13.26 mm	(19)
					0.32–0.625 μg/mL	

a Chit, chitosan; f-Chit, fungal chitosan; BSA, bovine serum albumin; bPEI, branched polyethylenimine; NChit, nanochitosan; AgNCs, silver nanoclusters; AuAgNCs, gold–silver nanoclusters.

### Susceptibility to Suture-Coated P-AgNPs

**Figure 5:**
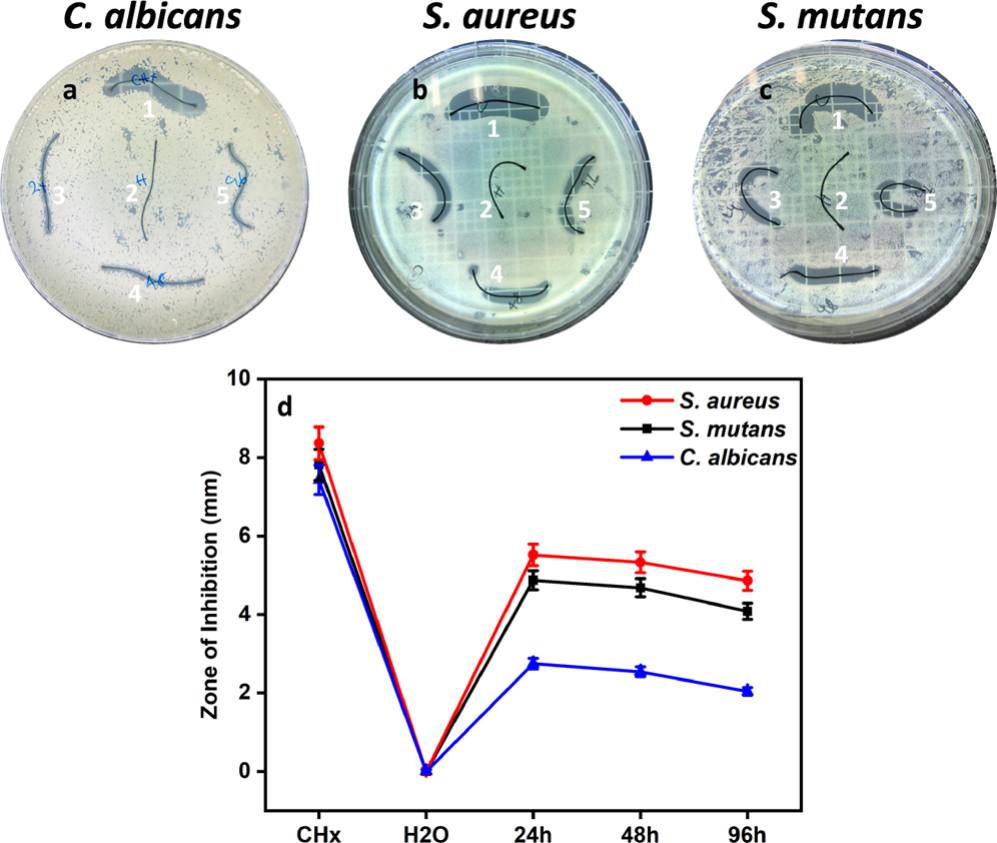
Antimicrobial effect of suture coated with P-AgNPs, which is represented in (a) *C. albicans*, (b) *S. aureus*, and (c) *S. mutans* for 24 h and (d) graphical representation of ZOI values.

Based on the promising results from the above-mentioned studies on P-AgNPs, suture-coated NPs were determined by placing a suture in the agar for 24 h (Figure 5a–c). An enhanced effect was observed in bacteria, whereas *Candida* showed major resistance. As expected, the CHx-coated fragments showed growth inhibition compared to P-AgNPs. Specifically, the suture incubated for 24 h with NPs showed higher inhibition than those incubated for 48 and 96 h. *S. aureus* was majorly inhibited with a ZOI of 4.86–5.52 mm, confirming that the NPs interact differently with each microorganism. Graph 5d and Table 3 clearly show the differences in the ZOI with individual microorganisms, explaining the impact of NPs on the inhibition of microbial growth.

**Table 3:** Numerical Representation of ZOI Values of Antimicrobial Sutures

**samples**	**suture-coated NPs ZOI (in mm)**		
	***S. mutans***	***S. aureus***	***C. albicans***
CHx	7.81 ± 0.3905	8.36 ± 0.418	7.43 ± 0.3715
24 h	4.87 ± 0.2435	5.52 ± 0.276	2.75 ± 0.1375
48 h	4.68 ± 0.234	5.33 ± 0.2665	2.54 ± 0.127
96 h	4.08 ± 0.204	4.86 ± 0.243	2.04 ± 0.102

The obtained results were compared with those published in the last 5 years, where Mathew et al. synthesized AgNPs by photoreduction onto silk sutures using an in situ method and tested against *S. mutans* and *S. aureus* for 7 days. The ZOI for the first and seventh days for *S. mutans* was 7 mm and for *S. aureus* was 8 mm, which did not show any increase in the ZOI even after a week of incubation.^51^ Basov et al. used AgNPs synthesized using PVP to coat various suture materials and to study the effect of cyclic freezing for deposition. Considering silk sutures, the 10-cyclic freezing process enhanced the antimicrobial effect by approximately 1.5 times with and without the cyclic freezing process against *E. coli*.^42^

Selvaraju et al. prepared propolis-extract-mediated AgNPs and coated catgut sutures using a slurry dipping technique. The authors observed clear evidence of bacterial inhibition by NP-coated sutures in *E. coli* and *S. aureus*.^18^ Syukri et al. used the *Eucalyptus camaldulensis* leaf extract as a capping and reducing agent for AgNP synthesis and coated the silk suture. From the suture-coated antimicrobial assay, the authors determined a 99% reduction in various Gram-positive and Gram-negative bacteria, including both ATCC strains and clinical isolates, including *S. aureus* and *E. coli*.^16^

Baygar et al. synthesized AgNPs using the bacterial extract of *Streptomyces* sp. AU2. Before the suture was coated, the suture surface was modified using propolis, and NPs were deposited using an in situ method. The authors analyzed the antimicrobial effect of multiresistant bacteria of *E. coli* and *S. aureus*, which are responsible for nosocomial infections, and observed a growth inhibition area of suture-coated AgNPs.^52^ Guadarrama-Reyes et al. fabricated AgNPs using *Chenopodium ambrosioides* and coated catgut and silk sutures. Then, the antibacterial effect of *S. aureus* and *E. coli* was seen, where both sutures exhibited enhanced inhibition. *S. aureus* showed 2.53 and 2.6 mm for silk- and catgut-coated sutures, respectively. In addition, *E. coli* expressed 2.46 mm inhibition for the suture types, with no significant differences.^53^

The above-mentioned data relate to our results, but most importantly, the concentration, surface charge, and coating method are all important. Therefore, in this study, we employed less than 6 μg/mL, a cationic and facile ex situ approach, to have an augmented antimicrobial suture effect within 24 h without any time-consuming process. The inhibitory effect was in the order *S. aureus* > *S. mutans* > *C. albicans*. This pattern is based on various factors and synergistic mechanisms, as explained in the following section.

### Antimicrobial Mechanisms

The antimicrobial effects of both P-AgNPs and suture-coated P-AgNPs inhibited bacterial and fungal growth. Thus, based on the Standard SNV 195920–1992, the NPs used in this work were demonstrated to have good antimicrobial potential as the ZOI was >1 mm. Therefore, in this section, the basis of this antimicrobial mechanism is explained. In this study, the developed P-AgNPs can provide a synergistic effect, which is due to the inherent antimicrobial properties of PEI and AgNPs.

In recent years, natural and synthetic polymers with intrinsic antimicrobial properties have been widely studied and have attracted significant interest from researchers.^54^ Specifically, cationic polymers are often employed to coat or functionalize nano- or biomaterials to inhibit microbial growth without the use of other chemical compounds, thereby reducing cytotoxicity. Regarding PEI, there are very few studies in which it was used for both reducing and stabilizing NPs. Thus, it not only provides a high positive charge to the NPs but also controls the size based on the optimum conditions that can be tuned. Therefore, the cationic charge prevents particle aggregation. In this study, we concluded that the antimicrobial suture effect is based on three characteristics of P-AgNPs.

*Cationic:* PEI enhances docking spontaneously or self-assembles onto cellular surfaces when compared to anionic particles. This increases the membrane diffusion and favors the adhesion to negatively charged nucleic acids, thereby instantly causing conformational changes, disruption by the ROS (reactive oxygen species) release and affecting various pathways^47^

*Size*: Owing to its smaller size, the surface area is increased, which in turn enhances the interaction with the microbial cell membrane and thereby releases silver ions.^16^ Specifically, NPs can penetrate the membrane via porins and completely disrupt cell physiology completely.^55^

*Internalization*: Both the above factors, such as positive charge and smaller size of NPs, play a vital role in the diffusion of NPs inside microorganisms.

The primary function of Ag ions is to initiate ROS production, which initiates biocidal or fungicidal effects. This sets off a chain reaction that targets the cell membrane, leading to delamination and degradation. Additionally, it disrupts signaling and metabolic pathways as well as membrane and transport proteins. Ultimately, this causes replication failure, leading to microbial cell lysis and death.^56^

### Cell Viability

**Figure 6:**
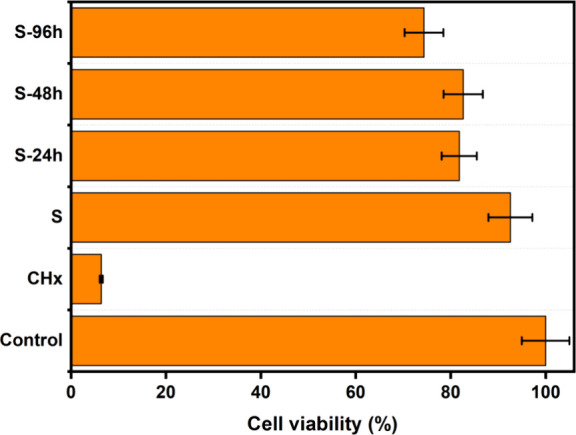
SCAPs cell viability was presented for NP-coated and control sutures.

The viability of SCAPs was tested in the presence of sutures for 24 h. As shown in Figure 6, the viability was greater than 75% for all samples (24, 48, and 96 h) when compared with the control group without any coating. The results obtained in this study are in good agreement with those reported in previous studies. For instance, in one of the studies, the authors coated PLGA sutures by the photodeposition of AgNPs and tested their cytotoxicity in 3T3 murine fibroblasts. Toxicity was assessed for approximately a week, and it was concluded that there was no significant difference when compared with the control sutures.^45^

In another study, the biosynthesis of AgNPs was carried out using *Streptomycessp*. AU2 and coated with PLGA sutures. When tested on 3T3 murine fibroblast cells for a prolonged period of 10 days, it did not show any effect or negative impact on cell viability.^15^ In a recent study, AgNPs were biosynthesized by using *Eucalyptus camaldulensis* and coated with silk sutures. Later, the cytotoxicity on HaCaT keratinocytes for 7 days and the results showed that the suture-coated AgNPs showed more than 64% cell viability, indicating a significant effect on the cells depending on the incubation time with cells.^16^ These results show that the AgNP-coated sutures do not induce significant adverse effects on fibroblast cell lines with mild cell toxicity, proving that antimicrobial sutures can be used for biomedical applications.

## Conclusions

The management and treatment of SSI have become a major challenge and an expensive process. Therefore, the Centers for Disease Control and Prevention (CDC) has implemented some precautionary measures before, during, and after surgery to avoid SSI. Therefore, antimicrobial sutures have recently gained interest as indispensable features of sutures. Therefore, our findings demonstrated that PEI can be effectively used to produce spherical, cationic, and stable AgNPs. The silk suture with the NPs coating did not affect the inherent characteristics and showed significant antibacterial and antifungal properties, as evidenced by the distinguished ZOI with less toxicity to SCAP. Finally, this methodology avoids the use of strong and toxic reducing agents, which provide long-term stability. To gain more in-depth insights, more studies are needed to understand the inhibition mechanism at the cellular level. Thus, it is understandable that the antimicrobial sutures produced in this study are effective against SSI-based pathogens and can be developed as an alternative in surgical practice. Therefore, avoiding or reducing the treatment cost and patient morbidity against surgical wound-related microbial infections.
